# Polygenic association between attention-deficit/hyperactivity disorder liability and cognitive impairments

**DOI:** 10.1017/S0033291720005218

**Published:** 2022-10

**Authors:** Isabella Vainieri, Joanna Martin, Anna-Sophie Rommel, Philip Asherson, Tobias Banaschewski, Jan Buitelaar, Bru Cormand, Jennifer Crosbie, Stephen V. Faraone, Barbara Franke, Sandra K. Loo, Ana Miranda, Iris Manor, Robert D. Oades, Kirstin L. Purves, J. Antoni Ramos-Quiroga, Marta Ribasés, Herbert Roeyers, Aribert Rothenberger, Russell Schachar, Joseph Sergeant, Hans-Christoph Steinhausen, Pieter J. Vuijk, Alysa E. Doyle, Jonna Kuntsi

**Affiliations:** 1 Social, Genetic and Developmental Psychiatry Centre, Institute of Psychiatry, Psychology and Neuroscience, King's College London, London, UK; 2 MRC Centre for Neuropsychiatric Genetics & Genomics, School of Medicine, Cardiff University, Cardiff, UK; 3 Department of Psychiatry & Department of Environmental Medicine, Public Health at the Icahn School of Medicine at Mount Sinai, New York, NY, USA; 4 Department of Child and Adolescent Psychiatry, Central Institute of Mental Health and Mannheim Medical Faculty, University of Heidelberg, Heidelberg, Germany; 5 Karakter Child and Adolescent Psychiatry University Center, Nijmegen, The Netherlands; 6 Department of Cognitive Neuroscience, Donders Institute for Brain, Cognition and Behavior, Radboud University Medical Centre, Nijmegen, The Netherlands; 7 Department of Genetics, Microbiology and Statistics, Faculty of Biology, University of Barcelona, Catalonia, Spain; 8 Centro de Investigación Biomédica en Red de Enfermedades Raras (CIBERER), Instituto de Salud Carlos III, Madrid, Spain; 9 Institut de Biomedicina de la Universitat de Barcelona (IBUB), Barcelona, Catalonia, Spain; 10 Institut de Recerca Sant Joan de Déu (IR-SJD), Esplugues de Llobregat, Catalonia, Spain; 11 Psychiatry, Neurosciences and Mental Health, The Hospital for Sick Children, University of Toronto, Toronto, Canada; 12 Departments of Psychiatry and Neuroscience and Physiology, SUNY Upstate Medical University, Syracuse, New York, USA; 13 Departments of Human Genetics and Psychiatry, Donders Institute for Brain, Cognition and Behaviour, Radboud University Medical Centre, Nijmegen, The Netherlands; 14 Department of Psychiatry and Biobehavioral Sciences, Semel Institute for Neuroscience and Human Behavior and David Geffen School of Medicine at UCLA, Los Angeles, CA, USA; 15 Department of Developmental and Educational Psychology, University of Valencia, Valencia, Spain; 16 Geha Mental Health Center, Petah Tikva, Sackler School of Medicine, Tel-Aviv University, Tel-Aviv, Israel; 17 Department of Child and Adolescent Psychiatry and Psychotherapy, University of Duisburg-Essen, Essen, Germany; 18 Department of Psychiatry, Hospital Universitari Vall d'Hebron, Barcelona, Catalonia, Spain; 19 Group of Psychiatry, Mental Health and Addictions, Vall d'Hebron Research Institute (VHIR), Barcelona, Catalonia, Spain; 20 Biomedical Network Research Centre on Mental Health (CIBERSAM), Barcelona, Catalonia, Spain; 21 Department of Psychiatry and Forensic Medicine, Universitat Autònoma de Barcelona, Barcelona, Catalonia, Spain; 22 Department of Genetics, Microbiology, and Statistics, Faculty of Biology, University of Barcelona, Catalonia, Spain; 23 Department of Experimental Clinical and Health Psychology, Ghent University, Gent, Belgium; 24 Department of Child and Adolescent Psychiatry and Psychotherapy, University Medical Center Göttingen, Goettingen, Germany; 25 Department of Clinical Neuropsychology, Vrije Universiteit, Amsterdam, The Netherlands; 26 Department of Child and Adolescent Psychiatry, University of Zurich, Zurich, Switzerland; 27 Clinical Psychology and Epidemiology, Institute of Psychology, University of Basel, Basel, Switzerland; 28 Department of Child and Adolescent Psychiatry, University of Southern Denmark, Odense, Denmark; 29 Centre of Child and Adolescent Mental Health, Capital Region Psychiatry, Copenhagen, Denmark; 30 Center for Genomic Medicine, Massachusetts General Hospital, Boston, MA, USA; 31 Massachusetts General Hospital and Harvard Medical School, Boston, MA, USA

**Keywords:** ADHD, attention, cognition, inhibition, polygenic risk scores, reaction time variability

## Abstract

**Background:**

A recent genome-wide association study (GWAS) identified 12 independent loci significantly associated with attention-deficit/hyperactivity disorder (ADHD). Polygenic risk scores (PRS), derived from the GWAS, can be used to assess genetic overlap between ADHD and other traits. Using ADHD samples from several international sites, we derived PRS for ADHD from the recent GWAS to test whether genetic variants that contribute to ADHD also influence two cognitive functions that show strong association with ADHD: attention regulation and response inhibition, captured by reaction time variability (RTV) and commission errors (CE).

**Methods:**

The discovery GWAS included 19 099 ADHD cases and 34 194 control participants. The combined target sample included 845 people with ADHD (age: 8–40 years). RTV and CE were available from reaction time and response inhibition tasks. ADHD PRS were calculated from the GWAS using a leave-one-study-out approach. Regression analyses were run to investigate whether ADHD PRS were associated with CE and RTV. Results across sites were combined via random effect meta-analyses.

**Results:**

When combining the studies in meta-analyses, results were significant for RTV (*R*^2^ = 0.011, *β* = 0.088, *p* = 0.02) but not for CE (*R*^2^ = 0.011, *β* = 0.013, *p* = 0.732). No significant association was found between ADHD PRS and RTV or CE in any sample individually (*p* > 0.10).

**Conclusions:**

We detected a significant association between PRS for ADHD and RTV (but not CE) in individuals with ADHD, suggesting that common genetic risk variants for ADHD influence attention regulation.

## Introduction

A recent case-control genome-wide association study (GWAS) identified, for the first time, 12 independent loci significantly associated with attention-deficit/hyperactivity disorder (ADHD) (Demontis et al., 2019). This GWAS enables further genetic investigations using polygenic risk scores (PRS), which are calculated for each individual by computing the sum of their risk alleles across the genome, weighted by effect sizes (Choi, Mak, & O'Reilly, 2018). PRS provide an estimate of the genetic propensity to ADHD at the individual level that can be used to investigate shared genetic etiology between ADHD and other phenotypes.

Previous studies on general population samples show that ADHD PRS are associated with a wide range of psychiatric and somatic disorders and traits, such as depression, anxiety, neuroticism, irritability, childhood internalizing and externalizing symptoms, obesity-related phenotypes and smoking (Brikell et al., 2018; Du Rietz et al., 2018; Riglin et al., 2017). Only a few of these population-based studies explored the cognitive phenotypes associated with ADHD using polygenic approaches, but have provided initial evidence for an association between PRS for ADHD and lower general cognitive ability (Du Rietz et al., 2018; Martin, Hamshere, Stergiakouli, O'Donovan, & Thapar, 2015a), educational attainment (Stergiakouli et al., 2017) and working memory, but not inhibition impairments (measured with the Opposite Words Task; Martin et al., 2015a). Evidence from clinically diagnosed samples with ADHD remains even more limited. The findings reported to date indicate an association of ADHD PRS with low academic achievement (Vuijk et al., 2019) and poor working memory and arousal-alertness, measured with latent variables (Nigg et al., 2018). In contrast, no significant associations emerged between PRS for ADHD and latent variables capturing inhibition or speed of responses (Nigg et al., 2018). A recent study found that PRS for ADHD were associated with a measure of interference, the ‘variance of word interference time’ in the Stroop test (Chang, Yang, Wang, & Faraone, 2020).

We now extend, in a sample of 845 people with ADHD, the previous PRS investigations of ADHD-related cognitive phenotypes to two cognitive measures that have extensive evidence from phenotypic studies of a strong association with ADHD, but have not yet been investigated using PRS: increased reaction time variability (RTV) and commission errors (CE) (Kuntsi et al., 2010; Loo et al., 2009; Schachar et al., 2007; van Rooij et al., 2015). RTV captures the highly variable speed of responding that is strongly characteristic of people with ADHD across a variety of cognitive tasks requiring a fast response (Kofler et al., 2013; Kuntsi et al., 2013), and has been linked in EEG and skin conductance studies to attention allocation and peripheral hypo-arousal (Cheung et al., 2017; James, Cheung, Rijsdijk, Asherson, & Kuntsi, 2016). CE, which represent the responses to non-target stimuli on inhibitory tasks such as the Go/No-Go task, capture failures to withhold responding.

Family and twin studies suggest a significant degree of familial/genetic sharing between ADHD and both RTV and CE (Kuntsi et al., 2010, 2014). For example, in a large study of 1265 children and adolescents, including 464 participants with ADHD, we observed a familial correlation of 0.74 between ADHD and RTV, and 0.45 between ADHD and CE (Kuntsi et al., 2010). The analyses further indicated a significant degree of etiological separation in the association of ADHD with RTV and CE (Kuntsi et al., 2010), with a similar conclusion emerging also from model fitting analyses in a population twin sample of 1312 children (Kuntsi et al., 2014). Family model fitting analyses also showed a high familial correlation between RTV obtained from two different tasks (a four-choice reaction time task, the Fast task, and a Go/No-Go task; rf = 0.75) (Kuntsi et al., 2010), suggesting RTV can be combined across such tasks for further genetic investigations.

Using a polygenic approach, we can move beyond the inferred etiological sharing between ADHD and RTV or CE that rely on comparisons of related individuals (in twin and family designs), to test the associations using molecular genetic data in unrelated individuals. Specifically, in this collaborative study using ADHD samples from several international sites, we derive PRS for ADHD from the recent GWAS (Demontis et al., 2019) to test whether genetic variants that contribute to ADHD also influence the cognitive impairments captured by RTV and CE in people with ADHD.

## Methods

### Discovery sample

As the discovery dataset, we used the Psychiatric Genomics Consortium (PGC) and iPSYCH Danish data analyzed in the recently published GWAS of ADHD (Demontis et al., 2019). This GWAS consists of 11 studies, with a total of 19 099 ADHD cases and 34 194 control subjects of European ancestry (full sample sizes are given in online Supplementary Table S1).

### Target samples and cognitive assessments

From the above discovery sample, four sub-samples from different sites were used as target samples applying a leave-one-study-out approach: International Multisite ADHD Genetics Project (IMAGE-I, subdivided here to IMAGE-8 and IMAGE-Dutch that had different cognitive test batteries), University of California Los Angeles (UCLA), Toronto and Barcelona. All participants for each site completed a comprehensive protocol of cognitive tasks, which differed for each site. Participants from IMAGE-8 performed a four-choice reaction time task (Fast task) and a version of the Go/No-Go task with fast and slow conditions, whereas IMAGE-Dutch participants performed the Stop-Signal Task (SST). At UCLA and Barcelona, participants performed the Continuous Performance Test II (CPT-II), whereas the Go/No-Go task was administered in Toronto. Descriptive statistics for each sample are shown in Table 1. Based on previous publications, cognitive variables were selected from the tasks that showed a significant ADHD case-control difference (effect sizes ranging from 0.32 to 0.95 for RTV, and from 0.38 to 0.42 for CE; Alemany et al., 2015; Hale et al., 2014; Kuntsi et al., 2010; Schachar et al., 2007; van Rooij et al., 2015). RTV [standard deviation (s.d.) of reaction times] was obtained from each of the tasks. Evidence for comparability between tasks was previously obtained from model fitting analyses on the fast task and Go/No-Go task, which indicated a high familial correlation (rf = 0.75) between RTVs obtained from each task, suggesting they are measuring largely the same liability (Kuntsi et al., 2010). CE was obtained from the CPT-II and Go/No-Go tasks only. The high rates of Go-stimuli in the CPT-II task make this task comparable to a Go/No-Go task.

**Table 1.** Descriptive statistics for all samples

Sample	*N*	IQ mean (s.d.)	Age mean (s.d.)	Sex M:F
IMAGE-I				
IMAGE-8	143	103.78 (15.24)	11.30 (2.67)	231:26
IMAGE-Dutch	226	98.96 (11.52)	11.50 (2.47)	119:24
UCLA	55	113.33 (15.03)	11.43 (2.98)	30:25
Toronto	54	101.24 (11.40)	9.38 (2.12)	42:12
Barcelona	367	NA	33.24 (10.54)	249:118

IMAGE, International Multisite ADHD Genetics Project; UCLA, University of California Los Angeles.

#### IMAGE-I

*Sample:* IMAGE-I is a European project on ADHD familiality using a common protocol of centralized training and data management. IMAGE-I includes data from different European sites and Israel, recruited from specialist clinics in Tel-Aviv, Essen, Gottingen, Brussels, Dublin, Valencia, Zurich, London, Nijmegen and Amsterdam (Kuntsi, Neale, Chen, Faraone, & Asherson, 2006a; Müller et al., 2011a; Müller et al., 2011b). The full IMAGE-I sample consisted of 782 individuals with DSM-IV ADHD combined type (680 ADHD combined type probands including 102 of their siblings who also met criteria for ADHD) and 808 additional unaffected siblings aged 6–19 years (Kuntsi et al., 2006a). All participants were recruited from specialist clinics. In IMAGE-I, parents of children were interviewed by trained researchers with the Parental Account of Childhood Symptom (PACS), a semi-structured, standardized, investigator-based interview developed as an instrument to provide an objective measure of child behavior. Both parents and teachers completed the respective versions of the Conners' ADHD rating scales and the Strengths and Difficulties Questionnaire (SDQ). Exclusion criteria were autism, epilepsy, IQ < 70, brain disorders and any genetic or medical disorder associated with externalizing behaviors that might mimic ADHD. Wherever possible, families withdrew stimulant medication for 1 week prior to research assessments to allow for more accurate ascertainment of the current level of ADHD symptoms and behaviors. Alternatively, clinical interviews were based on medication-free periods. A minimum of a 48-h medication-free period was required for cognitive testing. All data were collected with informed consent of the parents and with the approval of the site's Institutional Review Board (IRB) or Ethical Committee.

Due to differences in the protocol of the cognitive tasks, IMAGE-I can be subdivided into two subsamples: IMAGE-8 (including participants from Tel-Aviv, Essen, Gottingen, Brussels, Dublin, Valencia, Zurich and London) and IMAGE-Dutch (including participants from Nijmegen and Amsterdam). In the current study, we included only participants with an ADHD diagnosis who had both cognitive and genetic data available. The final sample consisted of 143 ADHD participants from the IMAGE-8 study and 226 ADHD participants from the IMAGE-Dutch study.

*Tasks: Fast-Task, Go/No-Go and SST:* The Fast task is a computerized four-choice reaction time (RT) task which measures performance under a baseline (slow-unrewarded) and a fast-incentive condition (Andreou et al., 2007; Kuntsi et al., 2006b). In the current study, only data from the baseline condition was included as this condition is more sensitive to ADHD (Kuntsi et al., 2013). The baseline condition consisted of 72 trials. Four empty circles (warning signals, arranged horizontally) first appeared for 8 s, after which one of them (the target) was colored in. Participants were asked to press the response key that corresponded to the position of the target. Following a response, the stimuli disappeared from the screen and a fixed inter-trial interval of 2.5 s followed. Speed and accuracy were emphasized equally.

The Go/No-Go task is a computerized test used to assess inhibitory control (Börger & van der Meere, 2000; Kuntsi, Andreou, Ma, Börger, & van der Meere, 2005; van der Meere, Stemerdink, & Gunning, 1995). On each trial of the Go/No-Go task, one of two possible stimuli appeared for 300 ms in the middle of the computer screen. The child was instructed to respond only to the Go stimuli and to withhold their response to No-Go stimuli. Participants were asked to react as quickly as possible while maintaining a high level of accuracy. The proportion of Go stimuli to No-Go stimuli was 4:1. This version of the Go/No-Go task consisted of three conditions (slow, fast and incentive). Here, we use data only from the slow condition, which show a strong association with ADHD (Andreou et al., 2007; Kuntsi, Wood, Van Der Meere, & Asherson, 2009; Uebel et al., 2010). The slow condition consisted of 72 trials and were presented with a fixed inter-stimulus interval of 8 s.

The SST is a response inhibition task, where participants had to respond as quickly as possible to a Go stimulus by left or right button press, unless shortly after presentation it was followed by a Stop signal, in which case they were to withhold their response (25% of trials) (Logan, Cowan, & Davis, 1984). The task difficulty was adaptive, meaning delays between the Go and Stop stimulus were adjusted by 50 ms after every failed or successful response, leading to an approximate 50% success rate on the Stop-trials for all participants. The task consisted of two practice blocks and four test blocks, each consisting of 60 trials.

#### UCLA

*Sample:* At UCLA, 156 participants with ADHD were recruited as part of the PUWMa collaboration [Pfizer-funded study from the University of California, Los Angeles (UCLA), Washington University, and Massachusetts General Hospital (MGH)], which included 540 children and adolescents aged 5–18 years, and 519 of their parents, ascertained from 370 families with ADHD-affected sibling pairs. Children and adolescents were assessed according to DSM-IV-TR criteria. Families were recruited through clinical referrals, schools and responses to advertisements (e.g. newsletters, community newspapers or flyers distributed at parent meetings in the greater Los Angeles area). Respondents without a previous diagnosis of ADHD were screened with the parent and teacher version of the Swanson, Nolan and Pelham Rating Scale, SNAP-IV (Swanson et al., 2012). After initial screening, children and adolescents were assessed by master's level clinical psychologists or highly trained interviewers using the Schedule for Affective Disorders and Schizophrenia for School-Age Children-Present and Lifetime version (K-SADS-PL), as well as a parent-completed Child Behaviour Checklist (CBCL) and Teacher Report Form. Participants were excluded if they were positive for any of the following: neurological disorder, head injury resulting in concussion, lifetime diagnoses of schizophrenia or autism or estimated IQ < 70. Participants on stimulant medication were asked to discontinue use for 24 h prior to their visit. The final sample with both cognitive and genetic data available consists of 55 ADHD cases.

*Task: CPT-II:* The CPT-II (Conners, 2000) is a 14-min computerized task that consisted of six blocks and three sub-blocks. Participants were required to press the space button on the keyboard whenever any letter except the letter ‘X’ appeared on the computer screen. The task consisted of 360 trials, including 36 presentations of the inhibition target (X). Targets (including ‘go’ targets: A, B, C, D, F, I, L, O, T) were presented in randomized order for 250-ms with variable inter-trial interval of 750, 1750 and 3750 ms. The presentation order of the different inter-trial intervals varied between blocks. The Go:No/Go ratio was 9:1.

#### Barcelona

*Sample:* The Spanish sample included 607 adults with ADHD (age range 18–40 years), recruited and evaluated at the Hospital Universitari Vall d'Hebron in Barcelona. The diagnosis of ADHD was evaluated by clinicians with the Structured Clinical Interview for DSM-IV Axis I and II Disorders (SCID-I and SCID-II) and the Conner's Adult ADHD Diagnostic Interview for DSM-IV (CAADID Parts I and II). Exclusion criteria were IQ < 70, schizophrenia or other psychotic disorders, ADHD symptoms due to mood, anxiety, dissociative or personality disorders, adoption, sexual or physical abuse, birth weight <1.5 kg and other neurological or systemic disorders that might explain ADHD symptoms. Cognitive and genetic data were available from 367 ADHD participants. More information about this sample can be found elsewhere (Sánchez-Mora et al., 2015).

*Task: CPT-II:* See UCLA for task description.

#### Toronto

*Sample:* The initial Canadian ADHD sample included 248 children aged 6–16 years referred for ADHD, learning and/or behavioral problems to the Hospital for Sick Children, Toronto (Lionel et al., 2011). ADHD diagnostic information was obtained based on DSM-IV criteria from parents and teachers in semi-structured clinical interviews including the Parent Interview for Child Symptoms (PICS) and the Teacher Telephone Interview (TTI). The assessments were conducted by a social worker, a clinical nurse specialist or a clinical psychologist and supervised by a clinical psychologist or child psychiatrist. Exclusion criteria were an IQ < 80, pervasive developmental disorder, autism or comorbid psychiatric disorder that could better account for the disorder Participants who were treated with stimulant medication had to be unmedicated for a minimum of 24 h before assessment and testing. Cognitive and genetic data for this study were available from 54 children with ADHD.

*Task: Go/No-Go task:* This version of the Go/No-Go task involved 128 trials of which 32 were No-Go trials and 96 were Go trials. During the Go task, one of two possible letters was presented (an X or an O) on each trial. Participants were required to make a response to the Go task stimuli as quickly and as accurately as possible by pressing one key of a handheld response box for an X and the other for an O (Go stimuli). The No-Go task involved an auditory tone which was presented, at the same time as the stimulus (letters), at random, on 25% of trials. Participants were instructed to withhold their response when they heard the tone. The Go task stimulus was presented for 1000 ms immediately following a fixation point of 500 ms. The task included four blocks, each with 24 Go trials and eight No-Go Trials. The Go:No-Go ratio was 3:1.

### Data analyses

#### Quality control of genetic and cognitive data

Quality control of genetic data was previously performed and was available for analyses (for more information see Demontis et al., 2019).

To account for positive skewness of the cognitive data, we applied appropriate transformations to all cognitive measures for each variable prior to analyses. Square root transformations were used in all samples for CE. For RTV, we used a logarithm transformation for IMAGE-8 team, Dutch-IMAGE and UCLA, and square root transformation for Barcelona and Toronto. There were no extreme outliers for RTV or CE (>3.5 s.d.).

#### PRS analyses

The GWAS summary statistics used as the discovery sample included the four target sub-groups (IMAGE-I, Toronto, Barcelona and UCLA). For this reason, PRS were calculated from the main GWAS each time excluding one of the target samples using four leave-one-out association meta-analyses, to ensure entirely independent discovery and target samples. PRS were estimated for each target sample using PRSice-2 software (Euesden, Lewis, & O'Reilly, 2015) (https://www.prsice.info) and applying standard procedures (imputation quality cut-off using PRSice INFO >0.9, and minor allele frequencies cut-off using PRSice MAF > 0.05) (Choi et al., 2018). PRSice computes PRS by calculating the sum of trait-associated alleles, weighted by the log odds ratio generated from the discovery GWAS. An *R*^2^ ⩾  0.1 (250-kb window) including all single-nucleotide polymorphisms (SNPs) (*p*^1^, *p*^2^ = 1) was used for linkage disequilibrium (LD) clumping to keep a set of independent SNPs. Linear regression models were used to estimate associations between PRS and phenotypes in the target samples. PRS were calculated at a number of *p* value thresholds for SNP inclusion to provide the most predictive PRS. The *p* value thresholds used were 0.001, 0.05, 0.1, 0.2, 0.3, 0.4, 0.5 and 1. We included age, sex and the first five principal components (PCs) as covariates in all analyses, to control for population stratification. The number of PCs was chosen based on the cohort's sample size (all  <1000) in order to avoid overfitting and to reflect the differential power to capture true population structure by principal component analysis, as reported in Demontis et al. (2019). The estimated amount of variance explained by PRS (i.e. *R*^2^ values) that we report for each study are adjusted from a baseline model including the covariates; the reported regression coefficients and standard errors (s.e.) were standardized to have mean = 0 and s.d.  1 using the PRSice command (--score std). We performed stringent permutation testing within PRSice-2 using 10 000 permutations to control for type 1 error and to prevent data overfitting across the range of *p* value thresholds considered (0.001, 0.05, 0.1, 0.2, 0.3, 0.4, 0.5 and 1). The *p* values are reported before correction (indicated with ‘*p*’), and after correction (indicated with ‘empirical-*p*’). Online Supplementary Figs S1–S9 provides plots for the PRS prediction models for RTV and CE across all sites.

#### Meta-analyses

For the meta-analyses, we used a random effects model using the rma.uni function of the metafor package in R, with the method set to ‘REML’. Meta-analyses for both RTV and CE were performed across all samples at all thresholds to check the consistency of the associations between PRS and these measures (online Supplementary Tables S2 and S3). Combining all samples, the sample size for the meta-analysis consisted of *n* = 743 ADHD participants for RTV and *n* = 679 ADHD participants for CE.

## Results

### PRS in individual datasets

PRS for ADHD were not significantly associated with RTV in any of the individual datasets (*R*^2^ = 0.004, *p* = 0.771, empirical-*p* = 0.993, *β* = 0.024 for IMAGE-8; *R*^2^ = 0.016, *p* = 0.124, empirical-*p* = 0.317, *β* = 0.135 for IMAGE-Dutch; *R*^2^ = 0.008, *p* = 0.466, empirical-*p* = 0.823, *β* = 0.032 for UCLA; *R*^2^ = 0.031, *p* = 0.362, empirical-*p* = 0.459, *β* = 0.112 for Toronto; *R*^2^ = 0.012, *p* = 0.029, empirical-*p* = 0.079, *β* = 0.122 for Barcelona). All associations showed a positive direction. PRS for ADHD were not significantly associated and showed inconsistent direction of association with CE in any of the individual samples (*R*^2^ = 0.011, *p* = 0.085, empirical-*p* = 0.217, *β* = −0.104 for IMAGE-8; *R*^2^ = 0.036, *p* = 0.188, empirical-*p* = 0.556, *β* = 0.101 for UCLA; *R*^2^ = 0.013, *p* = 0.407, empirical-*p* = 0.761, *β* = −0.121 for Toronto; *R*^2^ = 0.006, *p* = 0.122, empirical-*p* = 0.301, *β* = 0.083 for Barcelona).

### Meta-analysis of all datasets

**Fig. 1.**
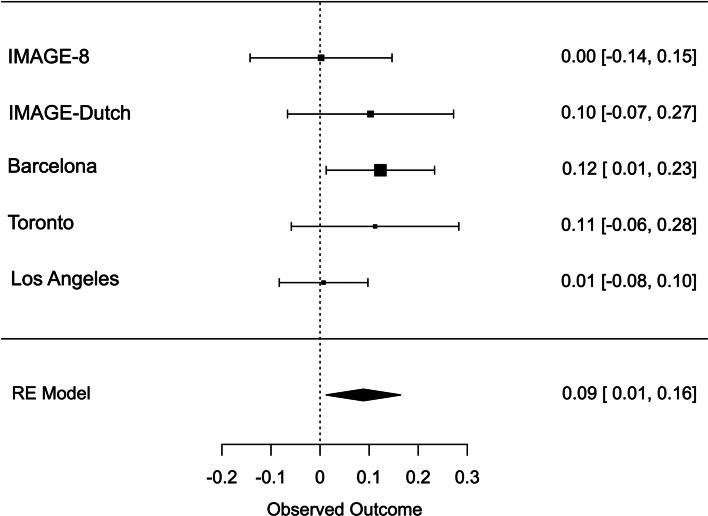
Forest plot of the meta-analysis of RTV. The overall estimate from random effects model is represented by the diamond below the individual study estimates.

**Fig. 2.**
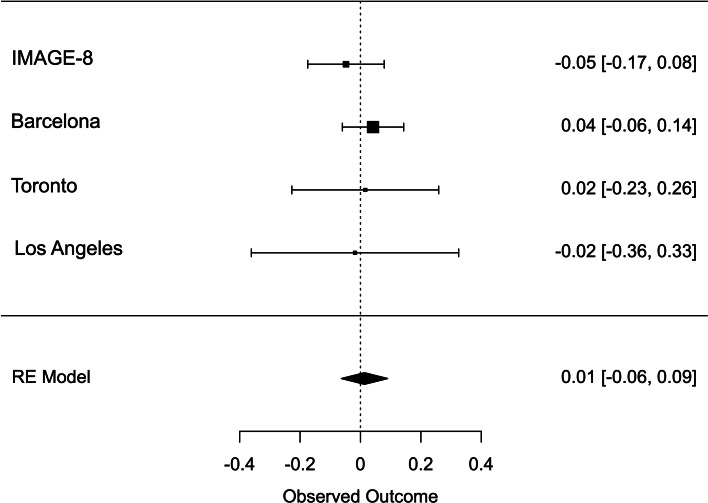
Forest plot of the meta-analysis of CE. The overall estimate from random effects model is represented by the diamond below the individual study estimates.

Meta-analysis across all thresholds for RTV showed that the best threshold for PRS association with RTV was 0.2 (online Supplementary Table S2). At this threshold, the PRS for ADHD was significantly associated with RTV (*R*^2^ = 0.011 *p* = 0.022, *β* = 0.088), with a positive direction. The best threshold for PRS association with CE was 0.001 (online Supplementary Table S3), but the association with CE did not reach significance CE (*R*^2^ = 0.011, *p* = 0.732, *β* = 0.013). Heterogeneity tests showed low heterogeneity across studies for both measures (*Q* = 3.777, *p* = 0.436, *I*^2^ = 13.513% for RTV; *Q* = 1.195, *p* = 0.754, *I*^2^ = 0% for CE). Forest plots for each variable are reported in Figs 1 and 2.

## Discussion

This is one of the largest studies investigating the association between ADHD PRS and cognitive impairments in individuals diagnosed with ADHD. Combining our samples in meta-analyses, our results show that polygenic risk for clinically diagnosed ADHD is positively associated with higher RTV, but not with CE as measured by Go/No Go tasks. These data suggest that common genetic variation relevant for ADHD influences attention regulation (RTV) but not response inhibition processes (CE) in a clinical ADHD sample. Whether the lack of an association with CE could reflect possible involvement of rare variants not detectable in this analysis or limited power to detect a potentially smaller association, requires further study.

Our results on RTV build on previous evidence from a smaller sample of children with ADHD showing a significant positive association between a latent variable of arousal-alertness and PRS for ADHD (Nigg et al., 2018). Of note, the association we observed between PRS for ADHD and RTV was mostly consistent across all *p* value thresholds in the meta-analysis, with only slight fluctuations in results possibly due to low power. Similarly, our results on CE are consistent with a previous population-based study and a clinical study showing no association between polygenic risk for ADHD and other inhibition measures (Martin et al., 2015a; Nigg et al., 2018), although a recent study did report an association between PRS for ADHD and interference when measured with the variance of word interference time in the Stroop test (Chang et al., 2020). Previous twin and sibling analyses have indicated a degree of shared genetic/familial influences on ADHD and response inhibition (Kuntsi et al., 2006b; Kuntsi et al., 2010). Further evidence from a sibling study suggested in fact two familial cognitive impairment factors for ADHD: a larger factor (85% of familial variance of ADHD) related to RTV, and a smaller factor (12.5% of familial variance of ADHD) capturing CE and omission errors (an overall measure of task accuracy) (Kuntsi et al., 2010). The findings from the sibling and twin studies (Kuntsi et al., 2010, 2014) suggested a potential separation, at the genetic level, between attention regulation and response inhibition processes in their association with ADHD. It is possible that our current analyses detected the larger factor accounted for by RTV in the sibling analyses (Kuntsi et al., 2010) while the smaller factor (accounting for CE) could not be detected with the current sample size. Future studies should investigate the genetic correlation between ADHD and RTV or CE across the whole genome using LD score regression, when summary statistics from GWAS on the appropriate cognitive traits will be available.

Although PRSs capture the common risk alleles that contribute to clinically diagnosed ADHD, they do not incorporate contributions from other genetic factors, such as copy number variants (CNVs) and single-nucleotide variants (SNVs) that may underlie the association of ADHD with RTV or CE. Several studies indicate a role for CNVs and SNVs in contributing to ADHD risk (Martin, O'Donovan, Thapar, Langley, & Williams, 2015b; Satterstrom et al., 2018; Thapar et al., 2016; Williams et al., 2012; Williams et al., 2010; Yang et al., 2013). CNVs were shown to be associated with cognitive features in the general population such as general cognitive ability (MacLeod et al., 2012), educational and occupational attainment (Kendall et al., 2017; Männik et al., 2015), and other cognitive phenotypes such as working memory, episodic memory, speed processing, visual attention and fluid intelligence (Kendall et al., 2017). Similarly, SNVs have been implicated in intellectual disability (Satterstrom et al., 2018). Yet, the extent to which CNVs and other genetic variants may contribute to cognitive impairments in individuals with ADHD is poorly understood and is an important direction for future research.

Although this is the largest study to date to investigate RTV and CE with a cutting-edge PRS method in a sample of individuals with clinically diagnosed ADHD, certain limitations need to be considered. First, our individual study analyses were underpowered due to the small sample sizes available in each single study. To increase statistical power, we analyzed the target studies with meta-analyses, reaching a combined sample size of *n* = 743 ADHD participants for RTV and *n* = 679 ADHD participants for CE; yet future studies, ideally with larger samples, are needed to replicate these results. Second, the age range of our participants was wide (8–45 years old). It would be informative in future larger studies to explore results separately for participants of different age groups (children, young adults and older adults). Third, our study included only participants of European ancestry; the generalizability of our findings to non-European populations requires further investigation. Fourth, the use of different tasks to reflect the two constructs of interest at different sites could have introduced heterogeneity in our data; however, we used random effects in the meta-analyses to account for between-study variation across sites. A further direction for future research is to widen the PRS investigation to additional cognitive impairments associated with ADHD.

Overall, polygenic risk associated with clinical ADHD diagnosis was associated with higher RTV in individuals with clinically diagnosed ADHD. Our results provide molecular genetic evidence that attention regulation and ADHD share common genetic factors. In other words, ADHD common variants not only contribute to risk of ADHD diagnosis, but are also a marker of poorer RTV performance in the context of having such a diagnosis. Further investigation, with bigger sample sizes, is needed to replicate these findings and to further determine the neurobiological mechanisms underlying this association. Furthermore, it is unknown whether the findings reported here are specific to ADHD or generalize to other disorders where increased RTV is also observed (such as bipolar disorder, schizophrenia and autism) (Brotman, Rooney, Skup, Pine, & Leibenluft, 2009; Kaiser et al., 2008; Karalunas, Geurts, Konrad, Bender, & Nigg, 2014).

## Supplementary material

For supplementary material accompanying this paper visit https://doi.org/10.1017/S0033291720005218. click here to view supplementary material
