# Supplementary material for: Polygenic association between attention-deficit/hyperactivity disorder liability and cognitive impairments
Source: Psychol Med. 2021 Feb 3;52(14):3150–8. doi: 10.1017/S0033291720005218 (PMC9693667; doi:10.1017/S0033291720005218)
Supplement: Supplementary file 1 [file S0033291720005218sup001.docx]

**Supplementary material**

**Polygenic association between attention-deficit/hyperactivity disorder ADHD liability and cognitive impairments**

*Isabella Vainieri, Joanna Martin, Anna Rommel, Philip Asherson, Tobias Banaschewski, Jan Buitelaar, Bru Cormand, Jennifer Crosbie, Stephen V. Faraone, Barbara Franke, Sandra Loo, Ana Miranda, Iris Manor, Robert D. Oades, Kirstin Purves, J. Antoni Ramos-Quiroga, Marta Ribasés, Herbert Roeyers, Aribert Rothenberger, Russell Schachar, Joe Sergeant, Hans-Christoph Steinhausen, Pieter J. Vuijk, Alysa E. Doyle, Jonna Kuntsi*

**Supplementary Tables**

**Table S1.** Total sample sizes for each of the PGC and iPSYCH studies of European Ancestry

| **Study** | **Number of cases** | **Number of controls** |
| --- | --- | --- |
| Bergen | 295 | 202 |
| Canada | 109 | 109 |
| Cardiff | 721 | 5081 |
| CHOP | 262 | 262 |
| Germany | 487 | 1290 |
| IMAGE-1 | 700 | 700 |
| IMAGE-2 | 624 | 1755 |
| PUWMa | 563 | 563 |
| Spain | 572 | 425 |
| Yale-Penn | 182 | 1315 |
| iPSYCH Danish | 14,584 | 22,492 |

Abbreviations: CHOP, Children’s Hospital of Philadelphia; IMAGE, International Multisite ADHD Genetics Project; PUWMa, Pfizer-funded study from the University of California, Los Angeles (UCLA), Washington University, and Massachusetts General Hospital (MGH).

**Table S2.** Meta-analysis results across all thresholds in all target samples for reaction time variability (RTV) controlling for age and sex

| **Threshold** | **SNP** | **R^2^** | **beta** | **p** | **CI (95%)** | **SE** | **i2** | **Q** | **Q-p** |
| --- | --- | --- | --- | --- | --- | --- | --- | --- | --- |
| 0.001 | 1069 | 0.006 | 0.082 | 0.025 | 0.010; 0.153 | 0.036 | 7.055 | 4.001 | 0.405 |
| 0.05 | 14361 | 0.005 | 0.068 | 0.062 | -0.002; 0.140 | 0.036 | 0 | 1.146 | 0.886 |
| 0.1 | 22858 | 0.006 | 0.063 | 0.083 | -0.008; 0.135 | 0.036 | 0 | 2.065 | 0.724 |
| 0.2 | 35793 | 0.011 | 0.088 | 0.022 | 0.012; 0.163 | 0.038 | 13.513 | 3.777 | 0.436 |
| 0.3 | 45909 | 0.003 | 0.074 | 0.062 | -0.003; 0.151 | 0.039 | 18.269 | 3.844 | 0.427 |
| 0.4 | 54170 | 0.004 | 0.064 | 0.085 | -0.008; 0.136 | 0.037 | 8.231 | 3.366 | 0.498 |
| 0.5 | 61172 | 0.001 | 0.066 | 0.078 | -0.007; 0.149 | 0.037 | 8.329 | 3.314 | 0.506 |
| 1 | 80663 | 0.004 | 0.069 | 0.056 | -0.002; 0.141 | 0.036 | 2.796 | 3.023 | 0.553 |

Abbreviations: single nucleotide polymorphisms, (SNPs); confidence intervals, (CI); standard errors, (SE).

**Table S3.** Meta-analysis results across all thresholds in all target samples for commission errors (CE) controlling for age and sex.

| **Threshold** | **SNP** | **R^2^** | **beta** | **p** | **CI (95%)** | **SE** | **i2** | **Q** | **Q-p** |
| --- | --- | --- | --- | --- | --- | --- | --- | --- | --- |
| 0.001 | 1069 | 0.011 | 0.013 | 0.732 | -0.063; 0.089 | 0.039 | 0 | 1.195 | 0.754 |
| 0.05 | 14361 | 0.009 | 0.005 | 0.886 | -0.076; 0.088 | 0.041 | 0 | 2.575 | 0.461 |
| 0.1 | 22858 | 0.010 | 0.009 | 0.869 | -0.099; 0117 | 0.055 | 28.527 | 3.567 | 0.312 |
| 0.2 | 35793 | 0.012 | 0.012 | 0.833 | -0.12; 0.149 | 0.068 | 49.137 | 5.823 | 0.123 |
| 0.3 | 45909 | 0.017 | 0.012 | 0.869 | -0.118; 0.139 | 0.065 | 46.002 | 5.271 | 0.152 |
| 0.4 | 54170 | 0.011 | 0.011 | 0.863 | -0.119; 0.142 | 0.066 | 46.621 | 5.381 | 0.145 |
| 0.5 | 61172 | 0.010 | 0.004 | 0.948 | -0.125; 0.133 | 0.065 | 44.953 | 5.104 | 0.164 |
| 1 | 80663 | 0.009 | 0.005 | 0.936 | -0.128; 0.139 | 0.068 | 47.217 | 5.378 | 0.146 |

Abbreviations: single nucleotide polymorphisms, (SNPs); confidence intervals, (CI); standard errors, (SE).

**Supplementary Figures**

**Figure S1.** Plot for reaction time variability (RTV) for the International Multisite ADHD Genetics Project (IMAGE) - 8

**Figure S2.** Plot for reaction time variability (RTV) for the International Multisite ADHD Genetics Project (IMAGE) - Dutch

**Figure S3.** Plot for reaction time variability (RTV) for Los Angeles

**Figure S4.** Plot for reaction time variability (RTV) for Toronto

**Figure S5.** Plot for reaction time variability (RTV) for Barcelona

**Figure S6.** Plot for commission errors (CE) for the International Multisite ADHD Genetics Project (IMAGE) - 8

**Figure S7.** Plot for commission errors (CE) for Los Angeles

**Figure S8.** Plot for commission errors (CE) for Toronto

**Figure S9.** Plot for commission errors (CE) for Barcelona
